# Association of Substance Use With Trauma Outcomes Throughout the COVID-19 Pandemic at a New York City Level II Trauma Center

**DOI:** 10.7759/cureus.91330

**Published:** 2025-08-31

**Authors:** Kaylee Bressler, Madison Salvitti, Risa Kiernan, Dennis Lysov, Hayden Roberts, Jennifer Feliciano-Maguire, Jinky DeCastro Singson, Dmitriy Karev, Stephen Dirusso

**Affiliations:** 1 General Surgery, New York Institute of Technology College of Osteopathic Medicine, Old Westbury, USA; 2 General Surgery, Lankenau Medical Center, Wynnewood, USA; 3 General Surgery, Rutgers Robert Wood Johnson Medical School, New Brunswick, USA; 4 General Surgery, New York Institute of Technology College of Osteopathic Medicine, Jonesboro, USA; 5 Trauma, St. Barnabas Hospital Health System, New York City, USA

**Keywords:** coronavirus pandemic, covid-19, general trauma surgery, substance use trends, ­trauma

## Abstract

Introduction: Studies have demonstrated that the COVID-19 pandemic caused increased stress associated with higher substance abuse and mental health crises that disproportionately affected minority populations. A New York City (NYC) Level II trauma center registry was used to determine if there was an association between substance use, patient demographics, and injury severity measures for patients admitted before, during, one, and two years after the pandemic.

Methods: A retrospective review of 3,172 patients was conducted using the NYC Level II trauma center registry. Cohorts included PreCOVID (2019) (N=704), COVID (2020) (N=787), 1yrPostCOVID (one year post-COVID, 2021) (N=883), and 2yrsPostCOVID (two years post-COVID, 2022) (N=798). Demographics and injury severity measures were analyzed using IBM SPSS Statistics v27 (IBM Corp., Armonk, United States) with p<0.05 indicating significance.

Results: Substance use significantly increased from PreCOVID to COVID and stayed elevated 1yrPostCOVID before returning to PreCOVID levels by 2yrsPostCOVID. Both men and women had increased rates of admission with positive substance use during COVID; however, the men recovered faster, with levels returning close to pre-pandemic levels by 2yrsPostCOVID. The admissions of women with positive substance use remained increased for 2yrsPostCOVID. The age group that was most affected by positive substance use was 25-34 years, which had a significant 4% increase from PreCOVID to COVID, and then steadily declined over 1yrPostCOVID and 2yrsPostCOVID. Minority populations, including Black Hispanic (BH), Black Non-Hispanic (BNH), and White Hispanic (WH), had increased substance use during COVID and 1yrPostCOVID compared to White Non-Hispanic (WNH). Positive substance use related to penetrating trauma increased during COVID, stayed increased 1yrPostCOVID, and then returned to PreCOVID levels by 2yrsPostCOVID. Positive substance use related to injury severity score, New Injury Severity Score (NISS), and Glasgow Coma Score (GCS) showed variable trends throughout the COVID timeline.

Conclusion: Results indicate that the pandemic led to an increase in substance use-related trauma admissions during and one year after the pandemic, which predominantly affected minority populations. This finding is consistent with the many other disparities minority populations faced during the pandemic.

## Introduction

New York City (NYC) was one of the epicenters of the COVID-19 pandemic, with NYC Health reporting a total of 190,408 cases in the first two months, 43,252 of which were in the Bronx [1]. At the onset of the pandemic, state governments initiated stay-at-home orders that were initially set to last two weeks, in hopes of stopping the spread of the virus. As the number of cases grew, so did the length of the isolation order. In NYC, the lockdown lasted three months before the city began phase one of its four-phase reopening plan [2]. These lockdown orders isolated people inside their homes with minimal in-person interactions. In a systematic review, Roberts et al. reported an overall trend of increased substance use during the COVID-19 pandemic, with mental health factors being highly correlated to this increase [3]. 

The Surgeon General's office warns how alcohol and drugs alter one’s brain chemistry, resulting in lower inhibition, increased impulsivity, and poor judgment, leading to increased risk-taking behavior, which may result in serious injuries [4]. Existing studies done by Cowperthwaite and Burnett (2011) and Lucas (2005), prior to the pandemic, have described higher complication rates and longer hospital stays for trauma patients who had a positive substance use test [5,6]. Few studies have examined patterns of substance use in trauma during and after the pandemic. Additionally, variations in the number of COVID-19 cases and state lockdown procedures may influence the amount of drug and alcohol use seen at specific trauma centers. 

The objective of this study was to evaluate rates of substance use, as well as patient and injury characteristics, among trauma patients throughout the COVID-19 pandemic at a NYC hospital. The study findings may help guide resource allocation and public health initiatives in the event of another pandemic and lockdown. 

## Materials and methods

This study was a retrospective analysis of prospectively collected data from St. Barnabas Hospital, a Level II COVID-19 epicenter trauma center in the Bronx, NYC. Institutional Review Board approval was previously obtained for data collection and the use of the trauma registry for research. 

The time period from March 20th to December 13th was used to compare data across the years 2019 to 2022. These dates correspond to the NYC lockdown implementation and the beginning of reopening during the initial wave of COVID-19. The cohorts were: PreCOVID (2019), COVID (2020), 1yrPostCOVID (one year post-COVID, 2021), and 2yrsPostCOVID (two years post-COVID, 2022). De-identified data were extracted for all trauma admissions during these time periods, including demographics, mechanism of injury (MOI), injury severity measurements, and discharge dispositions. PreCOVID was used as the reference group, and all comparisons were made against this cohort. 

Variables

Demographic information included sex, race and ethnicity, age, and insurance payor status. Race and ethnicity variables were combined into a single RaceEthnicity variable to better reflect the diversity of our patient population. The registry race variable comprised: American Indian, Asian, Black, Native Hawaiian or Other Pacific Islander, White, Not Applicable, Other, or Unknown. The registry ethnicity variable comprised: Hispanic, Non-Hispanic, Not Applicable, or Unknown. Patients who identified racially or ethnically as "Not Applicable" or "Unknown" could not be reasonably interpreted and were excluded. The RaceEthnicity variable included five groups of patients who identified as a combination of Black Hispanic (BH), Black Non-Hispanic (BNH), White Hispanic (WH), White Non-Hispanic (WNH), and Other. The "Other" group was composed of all other races and ethnicities grouped together due to limited numbers in the registry. 

A substance use variable was created using the drug and alcohol screen results. Patients with a positive drug and/or alcohol screen were classified as positive for substance use. Those who were not tested or had negative results were classified as negative. The current protocol at this institution is to only test if there's a clinical suspicion of substance use on presentation, so those who were not tested were presumed to be clinically sober at the time of examination. 

Trauma injury severity measures included emergency department (ED) activation level, New Injury Severity Score (NISS) [7], Glasgow Coma Score (GCS) [8], Revised Trauma Score (RTS) [9], shock index (SI) [10], base excess (BE) [11], and ICU length of stay (LOS). 

MOI was categorized as blunt, penetrating, or other, derived from International Classification of Diseases, 10th Revision (ICD-10) codes [12]. Blunt injuries included motor vehicle collisions (MVC), falls, cyclists/pedestrians struck, and other. Penetrating injuries included firearm-related and other penetrating injuries. The "other" category included burns, animal wounds, and explosion injuries.

Statistical analyses

IBM SPSS Statistics v27 (IBM Corp., Armonk, United States) was used to perform all statistical analyses. Descriptive statistics were reported as absolute counts and percentages. Categorical variables were compared using the chi-square test, using PreCOVID as the reference category and comparing all other categories to it individually. Means of continuous variables were compared using the Student’s t-test. All proportions were analyzed using a two-proportion Z-test. All p-values were reported as two-sided, and results with p-values <0.05 were considered statistically significant.

## Results

Demographics

Table 1 outlines the demographics of patients with a positive substance use test included in this study. A total of 3,172 patients were analyzed, of whom 1,358 (42.8%) tested positive for substance use. There was a significant increase in positive tests during COVID (N=364, 46.3%) and 1yrPostCOVID (N=402, 45.5%) compared to PreCOVID (N=259, 36.8%), which then returned to PreCOVID levels during 2yrsPostCOVID (N=333, 41.7%).

**Table 1:** Positive substance use and demographics * indicates significance with p<0.05 when compared against the reference range of PreCOVID. Total (n) refers to the number of patients with that variable.

Variable	PreCOVID (2019), n (%)	COVID (2020), n (%)	1yrPostCOVID (2021), n (%)	2yrsPostCOVID (2022), n (%)
Total (n)	704	787	883	798
Substance Use				
Positive test	259 (36.8)	364 (46.3)*	402 (45.5)*	333 (41.7)
Gender				
Male	228 (32.4)	306 (38.9)*	344 (39.0)*	272 (34.1)
Female	31 (4.4)	58 (7.4)*	58 (6.6)	61 (7.6)*
Age				
16-24 yo	38 (5.4)	52 (6.6)	42 (4.8)	35 (4.4)
25-34 yo	58 (8.2)	92 (11.7)*	97 (11.0)	73 (9.1)
35-44 yo	50 (7.1)	72 (9.1)	84 (9.5)	75 (9.1)
45-54 yo	34 (4.8)	53 (6.7)	70 (7.9)*	48 (6.0)
55-64 yo	48 (6.8)	51 (6.5)	79 (8.9)	51 (6.4)
≥65 yo	31 (4.4)	44 (5.6)	30 (3.4)	51 (6.4)
Race/Ethnicity				
Black Hispanic	36 (5.1)	70 (8.9)*	66 (7.5)	55 (6.9)
Black Non-Hispanic	118 (16.8)	159 (20.2)	226 (25.6)*	159 (19.9)
White Hispanic	29 (4.1)	52 (6.6)*	51 (5.8)	39 (4.9)
White Non-Hispanic	69 (9.8)	68 (8.6)	51 (5.8)*	73 (9.1)
Other	7 (1.0)	15 (1.9)	8 (0.9)	7 (0.9)
Insurance Payor Status				
Private	71 (10.1)	127 (16.1)*	144 (16.3)*	126 (15.8)*
Public	162 (23.0)	209 (26.6)	221 (25.0)	130 (16.3)*
Out-of-pocket	5 (0.7)	7 (0.9)	5 (0.6)	9 (1.1)
Unknown/Other	21 (3.0)	21 (2.7)	32 (3.6)	68 (8.5)*

Sex differences were observed. Both male and female patients showed increased positive substance use during COVID; however, by 2yrsPostCOVID, male patients had returned to PreCOVID levels, while female patients remained elevated. The highest substance use was seen in patients aged 25-34, which significantly increased during COVID (N=92, 12%) compared to PreCOVID (N=58, 8%). Patients aged 45-54 showed a significant increase during 1yrPostCOVID (N=70, 8%) compared to PreCOVID (N=34, 5%), which returned to baseline by 2yrsPostCOVID (N=48, 6%).

Minority populations - including BH, BNH, and WH - experienced significant increases in substance use at different points during the pandemic (COVID: BH and WH; 1yrPostCOVID: BNH), with rates returning to PreCOVID levels by 2yrsPostCOVID. Patients with private insurance and positive substance use increased significantly during COVID (N=127, 16.1%), 1yrPostCOVID (N=144, 16.3%), and 2yrsPostCOVID (N=126, 15.8%) compared to PreCOVID (N=71, 10.1%). 

Trauma severity

Table 2 details injury severity measures for patients with a positive substance use test, showing mixed results. The number of level 1 ED activations - the highest level - significantly increased during COVID (N=128, 16.3%), 1yrPostCOVID (N=141, 16.0%), and 2yrsPostCOVID (N=126, 15.8%) compared to PreCOVID (N=84, 11.9%). Level 2 ED activations were significantly higher during COVID (N=201, 25.5%) than PreCOVID (N=143, 20.3%) but returned to previous levels by 1yrPostCOVID (N=205, 23.2%). There were no significant changes in mean GCS, RTS, SI, or ICU LOS.

**Table 2:** Positive substance use and injury severity measures * indicates significance with p<0.05 when compared against the reference range of PreCOVID.

Variable				PreCOVID (2019), n (%)					COVID (2020), n (%)		1yrPostCOVID (2021), n (%)	2yrsPostCOVID (2022), n (%)
Emergency Department (ED) Activation				704					787		883	798
Level 1				84 (11.9)					128 (16.3)*		141 (16.0)*	126 (15.8)*
Level 2				143 (20.3)					201 (25.5)*		205 (23.2)	155 (19.4)
Level 3				30 (4.3)					35 (4.4)		56 (6.3)	52 (6.5)
None				2 (0.3)					0 (0.0)		0 (0.0)	2 (0.3)
Glasgow Coma Score (GCS)						686			776		858	766
13-15				226 (32.9)					317 (40.9)*		357 (41.6)*	292 (38.1)*
9-12				11 (1.6)					17 (2.2)		16 (1.9)	16 (2.1)
3-8				16 (2.3)					28 (3.6)		20 (2.3)	19 (2.5)
Mean				14.02 ± 2.6					13.89 ± 2.7		14.21 ± 2.3	14.07 ± 2.5
Revised Trauma Score (RTS)							638		759		806	719
0-2.99				2 (0.3)					0 (0.0)		0 (0.0)	1 (0.1)
3.00-5.99				12 (1.9)					24 (3.2)		21 (2.6)	14 (1.9)
6.00-7.84				219 (34.3)					329 (43.3)		347 (43.1)	292 (40.6)
Mean				7.57 ± 0.9					7.61 ± 0.7		7.63 ± 1.6	7.62 ± 0.8
Shock Index (SI)	514								614		632	585
0.50-0.70			100 (19.5)						167 (27.2)*		154 (24.4)*	154 (26.3)*
>0.70			101 (19.6)						144 (23.5)		159 (25.2)*	118 (20.2)
Mean			0.6763 ± 0.199						0.6858 ± 0.189		0.6939 ± 0.243	0.6959 ± 0.230
Base Excess (BE)		453								531	531	672
Normal (-2 ≤ x ≤ 2)					75 (16.6)				169 (31.8)*		192 (36.2)*	98 (14.6)
Mild (-6 < x < -2)					33 (7.3)				46 (8.7)		44 (8.3)	22 (3.3)*
Moderate/Severe (≤-6)					37 (8.2)				42 (7.9)		50 (9.4)	21 (3.1)*
Mean		-1.91 ± 5.70								-1.09 ± 4.55	-0.75 ± 5.03*	-0.43 ± 5.17*
New Injury Severity Score (NISS)		703								785	879	770
Trauma (≤15)		160 (22.8)								247 (31.5)	257 (29.2)	231 (30.0)
Major trauma (>15)		99 (14.1)								116 (14.8)	144 (16.4)	97 (12.6)
Mean		15.95 ± 12.8								14.43 ± 11.8	15.24 ± 12.4	13.21 ± 10.7
ICU Admissions Total								222	205		209	131
Positive substance use								99 (44.6)	121 (59.0)		133 (63.6)	73 (55.7)
ICU Length of Stay												
Average days (SD)						5.31 ± 8.7			4.36 ± 5.4		5.34 ± 7.4	5.58 ± 8.9

The number of patients with a GCS of 13-15 significantly increased during COVID (N=317, 40.9%), 1yrPostCOVID (N=357, 41.6%), and 2yrsPostCOVID (N=292, 38.1%) compared to PreCOVID (N=226, 32.9%). No clinically relevant patterns were observed in RTS, SI, or BE, as all group means remained within normal ranges throughout the timeline.

Mean NISS scores significantly decreased from PreCOVID (15.95 ± 12.8) to 2yrsPostCOVID (13.21 ± 10.7). The proportion of patients with less severe injuries (lower NISS) significantly increased during COVID (N=247, 31.5%), 1yrPostCOVID (N=257, 29.2%), and 2yrsPostCOVID (N=231, 30.0%) compared to PreCOVID (N=160, 22.8%). ICU admissions also significantly increased during COVID (N=121, 59.0%), 1yrPostCOVID (N=133, 63.6%), and 2yrsPostCOVID (N=73, 55.7%) compared to PreCOVID (N=99, 44.6%).

MOI patterns

The MOI patterns for trauma patients with a positive substance use test are described in Table 3. There was a significant increase in penetrating injuries during COVID (N=104, 13%) and 1yrPostCOVID (N=115, 13%) compared to PreCOVID (N=62, 9%), which then returned to baseline levels by 2yrsPostCOVID (N=77, 10%). No significant change was observed in the number of blunt injuries. 

**Table 3:** Positive substance use and mechanism of injury (MOI) * indicates significance with p<0.05 when compared against the reference range of PreCOVID. Total (n) refers to the number of patients with that variable.

MOI	PreCOVID (2019), n (%)	COVID (2020), n (%)	1yrPostCOVID (2021), n (%)	2yrPostCOVID (2022), n (%)
Total (n)	703	786	882	769
Blunt	193 (27.5)	250 (31.8)	271 (30.7)	233 (30.3)
Penetrating	62 (8.8)	104 (13.2)*	115 (13.0)*	77 (10.0)
Other	4 (0.6)	10 (1.3)	15 (1.7)	18 (2.3)

Outcomes

Patient discharge outcomes are listed in Table 4. The proportion of patients with positive substance use discharged home without support significantly increased during COVID (N=241, 30.7%), 1yrPostCOVID (N=296, 33.6%), and 2yrsPostCOVID (N=236, 30.6%) compared to PreCOVID (N=168, 23.9%). Conversely, those discharged home with support significantly decreased during 1yrPostCOVID (N=8, 0.9%) and 2yrsPostCOVID (N=5, 0.6%) compared to PreCOVID (N=15, 2.1%). There were no significant differences in mean discharge GCS throughout the pandemic.

**Table 4:** Positive substance use and outcomes * indicates significance with p<0.05 when compared against the reference range of PreCOVID.

Variable	PreCOVID (2019), n (%)		COVID (2020), n (%)	1yrPostCOVID (2021), n (%)	2yrsPostCOVID (2022), n (%)
Discharge Disposition	703		786	882	771
Home without support	168 (23.9)		241 (30.7)*	296 (33.6)*	236 (30.6)*
Home with support	15 (2.1)		22 (2.8)	8 (0.9)*	5 (0.6)*
Rehab, medical facility, or other hospital		36 (5.1)	58 (7.4)	58 (6.6)	38 (4.9)
Left against medical advice (AMA)	31 (4.4)		35 (4.5)	31 (3.5)	39 (5.1)
Deceased	9 (1.3)		8 (1.0)	8 (0.9)	10 (1.3)
Discharge Glasgow Coma Score (GCS)					
Average score (SD)	14.94 ± 0.8		14.85 ± 1.0	14.94 ± 0.4	14.92 ± 0.6

## Discussion

Identifying trends and changes in the trauma registry throughout the COVID-19 pandemic can help pinpoint the populations most at risk and better prepare hospitals for future lockdowns [13]. In this study, we showed an increase in substance use during the pandemic, which led to changes in demographics and types of trauma patients admitted to our center. This rise in substance use among trauma patients aligns with increases seen in the general population. For example, Boschuetz et al. found that regular drinkers reported increased frequency and amount of alcohol consumption during the pandemic, suggesting it was used as a coping mechanism [14]. Similarly, Taylor et al. reported that rates of alcohol and recreational drug use increased alongside self-isolation [15]. The high substance use rates in our trauma registry likely reflect the increased stress and isolation experienced during the pandemic, which persisted beyond the initial lockdown, remaining elevated through 2022.

Examining this stress-induced rise in substance use reveals that different patient subgroups were affected at different times. Both male and female patients showed increases, but male patients returned to baseline levels sooner, suggesting their stress may have been relieved earlier. Only two age groups showed significant changes: patients aged 25-34 increased their substance use during the COVID period, possibly due to sudden isolation and restrictions on socializing. Patients aged 45-54 showed increased use during 1yrPostCOVID, suggesting a delayed stress response; they may have coped initially but became burned out as the pandemic and isolation dragged on. 

Among racial and ethnic groups, minority populations - including BH, BNH, and WH patients - experienced increased substance use during COVID and 1yrPostCOVID. Many studies have documented racial, ethnic, and socioeconomic disparities during the pandemic, with these groups suffering higher rates of COVID-19 infection, hospitalization, ICU admission, and death [16-18], as well as worsened mental health with increased anxiety and depression [19]. Our findings extend this evidence, showing that these disparities included higher substance use rates, likely reflecting greater stress and isolation. Identifying these high-risk subgroups can help direct support and resources during periods of increased stress.

Understanding the MOI among these patients also informs injury prevention strategies. Our study found an increase in penetrating traumas associated with positive substance use during COVID and 1yrPostCOVID. We hypothesize two contributing factors: more time spent at home and outside, with less time being spent at work, and the impact of substance use on decision-making and behavior. Previous studies have reported similar rises in penetrating and violent injuries in various United States regions [20-22]. Additionally, poor decision-making is a hallmark of substance use disorders and can have lasting health consequences [23,24]. Despite more penetrating injuries, injury severity remained relatively stable. Although level 1 ED activations increased during the pandemic, suggesting more severe injuries, no clinically significant differences were found in GCS, RTS, SI, BE, or NISS. The heightened ED response during the COVID influx may have extended to trauma care, contributing to more level 1 activations.

During the pandemic, many nonessential programs transitioned to an online telemedicine platform [25,26]. This included general rehabilitation services and substance use disorder treatment centers [27,28]. At our institution, more patients with positive substance use tests were discharged home without general rehab services during the pandemic, likely reflecting this shift. Telemedicine use has since grown widely for both general and substance-related rehab programs. Sistad et al. reported that veterans were 2.6 times more likely to initiate substance use treatment when referred to telehealth during COVID-19 compared to in-person appointments pre-pandemic [29]. However, disparities in telehealth access persist. Lau et al. found that non-English speakers, Black and Latinx patients, and those in lower-income zip codes (<$50,000/year) were less likely to schedule telemedicine video visits [30]. Together, these findings highlight the need to allocate resources to minority populations to ensure equitable access to telemedicine during future lockdowns.

The limitations of our study include its retrospective design, single-institution setting, and focus on an urban, socioeconomically constrained population, which limits generalizability. However, the diversity of our patients highlights groups at high risk and in need of targeted resources. Future research should use national trauma databases to examine larger, more representative populations to assess differences in demographics, injury mechanisms, and injury severity. Another limitation was the presumption that patients who did not show signs of intoxication were not tested for substance use, and therefore, were included with those who had negative test results. At this institution, only patients with high clinical suspicion of substance use are given a drug and alcohol screen. Therefore, there is the risk that some not tested may have had a positive substance use test, deflating the results presented.

## Conclusions

The COVID-19 pandemic was a period of extreme uncertainty that led to increased stress and isolation, which, in turn, contributed to a rise in substance use - reflected in our trauma registry data. Evaluating patient demographics helps identify those at highest risk for disproportionate outcomes and supports targeted resource allocation during future stress-inducing events. Likewise, recognizing the increased prevalence of penetrating injuries during the pandemic can inform future injury prevention strategies. The pandemic has permanently altered society and the healthcare landscape. It is essential to learn from this experience by addressing health disparities, supporting minority populations, and strengthening public health systems to prevent future crises and ensure equitable healthcare for all. 
